# Understanding the population genetics of *Plasmodium vivax *is essential for malaria control and elimination

**DOI:** 10.1186/1475-2875-11-14

**Published:** 2012-01-10

**Authors:** Alicia Arnott, Alyssa E Barry, John C Reeder

**Affiliations:** 1 Centre for Population Health, Burnet Institute, Melbourne, Australia; 2 Division of Infection and Immunity, the Walter and Eliza Hall Institute for Medical Research, Parkville, Australia; 3 Department of Medical Biology, University of Melbourne, Parkville, Australia; 4 Department of Epidemiology and Preventative Medicine, Monash University, Melbourne, Australia

**Keywords:** *Plasmodium vivax*, Malaria, Population genetics, Microsatellites, Genetic diversity, Elimination

## Abstract

Traditionally, infection with *Plasmodium vivax *was thought to be benign and self-limiting, however, recent evidence has demonstrated that infection with *P. vivax *can also result in severe illness and death. Research into *P. vivax *has been relatively neglected and much remains unknown regarding the biology, pathogenesis and epidemiology of this parasite. One of the fundamental factors governing transmission and immunity is parasite diversity. An understanding of parasite population genetic structure is necessary to understand the epidemiology, diversity, distribution and dynamics of natural *P. vivax *populations. In addition, studying the population structure of genes under immune selection also enables investigation of the dynamic interplay between transmission and immunity, which is crucial for vaccine development. A lack of knowledge regarding the transmission and spread of *P. vivax *has been particularly highlighted in areas where malaria control and elimination programmes have made progress in reducing the burden of *Plasmodium falciparum*, yet *P. vivax *remains as a substantial obstacle. With malaria elimination back on the global agenda, mapping of global and local *P. vivax *population structure is essential prior to establishing goals for elimination and the roll-out of interventions. A detailed knowledge of the spatial distribution, transmission and clinical burden of *P. vivax *is required to act as a benchmark against which control targets can be set and measured. This paper presents an overview of what is known and what is yet to be fully understood regarding *P. vivax *population genetics, as well as the importance and application of *P. vivax *population genetics studies.

## *Plasmodium vivax*: a global health threat

Presently, 2.85 billion people globally are at risk of *Plasmodium vivax *malaria infection [1]. Worldwide the regional incidence of *P. vivax *has been increasing despite a decrease in *Plasmodium falciparum *cases [2-5]. The most geographically widespread of the six *Plasmodium *species that infect humans, *P. vivax *is a major health threat to huge populations throughout Asia, the Middle East and the Pacific [1], where approximately 80 to 90% of the global *P. vivax *burden is concentrated [6]. A significant number of *P. vivax *cases also occur throughout Central and South America, and East and South Africa [6]. Despite this, research into *P. vivax *malaria has been relatively neglected and much detail of the biology, pathogenesis and epidemiology of this parasite remains unknown. Traditionally, infection with *P. vivax *was thought to be benign and self-limiting and was not considered a research priority in comparison with the enormous burden of morbidity and mortality presented by *P. falciparum *[7,8]. Recent evidence is however challenging the long-held notion of the benign nature of *P. vivax *malaria, demonstrating that infection with *P. vivax *can also result in severe illness and death [9-14]. Indeed, the severe manifestations of *P. vivax *disease are very similar to those caused by *P. falciparum *and include cerebral malaria, acute respiratory distress, lung injury, renal failure, hepatic dysfunction, shock and death [12,14].

Lack of knowledge regarding the transmission and spread of *P. vivax *has been particularly highlighted in areas where malaria control and elimination programmes have made progress in reducing the burden of *P. falciparum*, yet *P. vivax *remains as a substantial obstacle [15]. The emergence and spread of drug resistant *P. vivax *is also of serious concern. Indeed, in the context of achieving malaria elimination targets, reports of primaquine resistance, the only available treatment to prevent relapse, is particularly worrisome [16,17]. One of the key problems undermining effective malaria control is a lack of understanding of the underlying *P. vivax *population structure and transmission dynamics. Population genetic studies are needed to define the diversity, distribution and dynamics of *P. vivax *populations, as parasite populations differ widely between locations, due to factors including prevalence, vector species, host genetics and a variety of environmental influences [18-21]. Mapping of global and local *P. vivax *population structure is essential prior to establishing goals for elimination and the rollout of interventions, and detailed knowledge of the spatial distribution, transmission and clinical burden of *P. vivax *is required to act as a benchmark against which control targets can be set and measured [1,19,22,23]. The Malaria Eradication Research Agenda (malERA) Consultative Group on Basic Science and Enabling Technologies recently reported that no campaign for malaria control or elimination can proceed without a comprehensive knowledge of disease epidemiology and host-parasite-vector interactions, and how these interactions are affected by intensified intervention measures [24].

## Genetic diversity of *Plasmodium vivax*

Within a malaria endemic area, multiple parasite clones can often co-infect the same host. To understand local population structure and genetic diversity, it is essential to be able to distinguish between distinct clones within the same infection as well as between infections. Not only useful in the context of molecular epidemiology studies, identifying and managing multiple clone infections may have additional public health implications, as increasing, or high levels of multiple clone *P. vivax *infections may drive increases in parasite virulence and fitness, as parasite clones compete for both resources within the infected host and survival against antimalarial interventions [25]. However, identifying, and distinguishing between clones can be difficult because the genetic diversity of *P. vivax *populations can vary significantly due to variations in malaria epidemiology in different regions [26,27]. Isolates obtained from distinct populations can either be genetically very diverse, so that multiple infections are easy to determine using a single molecular marker, or they can have quite low levels of diversity or even be clonal in the case of very low transmission or epidemics, which makes it more difficult to distinguish polyclonal from true monoclonal infections [28].

Genotyping to determine the multiplicity of infection (MOI) can be performed using either one or two markers that are extremely polymorphic, or a larger number of less polymorphic genome-wide markers. Currently, regions of the genome containing polymorphic repeat sequences, such as microsatellites or surface antigen genes, are the markers of choice for doing this. Microsatellite markers are short, tandem, one to six nucleotide repeats, found frequently throughout the genome and are typically, selectively neutral. Prior to the identification of microsatellites, earlier studies genotyped strains on the basis of polymorphic coding regions within parasite antigens such as the circumsporozoite protein (CSP) and the merozoite surface protein 3 alpha (MSP-3α), for which there are fewer alleles than the microsatellite markers [25,29]. Therefore, the number of clones per infection was most likely underestimated (Table 1).

**Table 1:** Global detection of multiple clone infections using molecular markers in *Plasmodium vivax *populations

Study region	Study [reference]	Year samples collected	Number of samples analysed	Marker	Proportion infections with multiple clones (%)	Mean MOI
**Asia**						

Thailand	Imwong *et al*. 2007 [27]	1992-1993	92	Microsatellites	30 - > 60	1.4
	Imwong *et al*. 2005 [30]	1995-1998	100	CSP + MSP1	26	1.29
				MSP3α	19.3	
				CSP + MSP3α	35.6	

Laos	Imwong *et al *2007 [27]	2001-2003	81	Microsatellites	30 - > 60	1.5

Vietnam	Van den Eede *et al*. 2010 [33]	1999-2000	69	Microsatellites	100	3.7

India	Prajapati *et al*. 2006 [34]	2000-2004	252	GAM-1	13.09	N/S
	Imwong *et al*. 2007 [27]	2003-2004	90	Microsatellites	10-40	1.2
	Kim *et al*. 2006 [35]	2003-2004	151	CSP, MSP1, MSP3α	10.6	N/S

Sri Lanka	Wickramara-chchi *et al*. 2010 [36]	1998-2000	201	MSP3α	13.8-20	N/S
	Karunaweera *et al*. 2008 [26]	2005	50	Microsatellites	9.1-60	N/S
	Gunawardena *et al*. 2010 [37]	2003-2008	140	Microsatellites	55	N/S

				MSP3α	6	N/S
Pakistan	Khatoon *et al*. 2010 [38]	[2007]	50	MSP3β	12	
				MSP3α + MSP3β	2	N/S
	Zakeri *et al*. 2010 [39]	2008	187	CSP, MSP-1, MSP3α	30	

China		2004	54: Anhui Province		5.6	N/S
	Yang *et al*. 2006	2005	31: Guizhou Province	MSP3β	0	
		2004	14: Guangxi Province		0	
	Zhong *et al*. 2011 [41]	2006-2008	140	MSP3α	7.6-14.3	N/S
				MSP3β	14.3-15	

Myanmar	Kim *et al*. 2010 [42]	2000	96	CSP	24.5	N/S
				CSP	24.1	N/S
	Moon *et al*. 2009 [43]	2004	349		MSP1	16.4
				MSP3α	21.6	
	Zhong *et a.l *2011 [41]	2006-2008	72	MSP3α	10.2	N/S
				MSP3β	16.4	
	Gunawardena *et al*. 2010 [37]	2007	167	Microsatellites	67.1	N/S

East Timor	Chen *et al*. 2007 [44]	2001	17	CSP, MSP-1, AMA-1	35	N/S

**Middle East**						

Iran	Zakeri *et al*. 2003 [45]	2000-2001	107	MSP-1	20	N/S
	Zakeri *et al*. 2006 [46]	2000-2003	374	CSP	12	N/S
	Zakeri *et al*. 2006 [47]	2000-2003	144	MSP3α	3.5	N/S
	Zakeri *et al*. 2010 [39]	2008	150	CSP, MSP-1, MSP3α	24.6	N/S

Uzbekistan	Severini *et al*. 2004 [48]	1999-2002	12 indigenous cases	MSP-1	8	N/S
			10 imported cases		10	

Afghanistan				CSP	6.4	
	Zakeri *et al*. 2010 [49]	2007	202	MSP-1	0	N/S
				MSP3α	3.5	

Turkey	Zeyrek *et al*. 2010 [50]	2007-2008	31	MSP-1	3.2	N/S

**Africa**						N/S

Ethiopia	Gunawardena *et al*. 2010 [37]	2006-2008	118	Microsatellites	73.70	N/S

**South & Central America**						

Mexico	Joy *et al*. 2008 [20]	1997-2005	234	Microsatellites	15.8	1.01

Peru	Sutton *et al*. 2009 [51]	2003-2004	186	MSP3α	26.3	2-3^a^
	Van den Eede *et al*. 2010 [28]	2006-2008	159	Microsatellites	11-70	1.1

Brazil	Ferreira *et al*. 2007 [2]	1999	74	Microsatellites	48 (1999)	N/S
		2004-2005			49 (2004-5)	
	Rezende *et al*. 2009 [52]	2003-2005	44	Tandem repeats	0-66	N/S
	Rezende *et al*. 2010 [53]	2003-2005	53	Microsatellites	32-57	N/S
	Storti- Melo *et al*. 2009 [54]	2003-2005	155	CSP	0-39.3	N/S
	Orjuela-Sanchez *et al*. 2009 [55]	2005-2007	77	Microsatellites	10.1-42.4	N/S

French Guiana				MSP-1 (57 samples)	12.3	N/S
	Veron *et al*. 2009 [56]	2005-2006	109	MSP3α (109 samples)	13.8	
				MSP-1 + MSP3α (57 samples)	21	

Guyana	Bonilla *et al*. 2006 [57]	2000	61	CSP	39.3	N/S

Colombia	Imwong *et al*. 2007 [27]	2001-2003	82	Microsatellites	10-40	1.1
	Cristiano *et al*. 2008 [58]	2006	55	MSP3α	36.4	N/S

Venezuela	Ord *et al*. 2005 [59]	1995-1997	58	MSP3α	10	N/S
	Leclerc *et al*. 2005 [60]	Not stated	39	MSP-1	0	N/A

**Oceania**						

Papua New Guinea	Henry-Halldin *et al*. 2011 [61]	2001-2003	703: Wosera region	CSP	36.8	N/S
			986: Mugil region		34.4	
	Mueller *et al*. 2002 [62]	2002	11	MSP3α	18	N/S
	Koepfli *et al*. 2009 [63]	2004-2005	108	Microsatellites	81.4	1-8
	Gomez *et al*. 2003 [64]	Not stated	89	pvMS1	4.5	N/S

^a^Mean MOI was not reported, shown is the observed COI range

N/S: Mean MOI was not reported; N/A: none of the samples tested were polyclonal

The population genetic structure of *P. falciparum *is closely associated with transmission intensity, hence population structure and diversity varies greatly according to geographical location, at least on a global scale [65,66]. *Plasmodium falciparum *populations in regions with low levels of transmission generally have low proportions of polyclonal infection high levels of linkage disequilibrium (LD) suggesting significant inbreeding and infrequent recombination. The inverse is also true, with parasite populations in high transmission areas characterized by a high proportion of multiple infections low levels of LD suggesting significant outcrossing and frequent recombination [27,65]. Few studies have been performed amongst sympatric populations of *P. vivax *and *P. falciparum*, however from what is known, there appears to be a distinctly different model of population structure for *P. vivax *compared to that of *P. falciparum *[2,25,27,37]. Using samples collected from a low transmission setting in rural Amazonia, Ferreira and colleagues reported higher genetic diversity and frequency of polyclonal infections for *P. vivax *compared to *P. falciparum *[2]. Interestingly, strong LD, and frequent replacement of predominant microsatellite haplotypes over time was also observed amongst the same *P. vivax *population [2]. The unique biology of *P. vivax *is likely to be responsible for the apparent paradox of multiple clone infection in a low transmission setting. The latent hypnozoite stage of the *P. vivax *lifecycle increases the likelihood of superinfection with a second clone, potentially resulting in the reactivation of heterologous hypnozoites and an increased likelihood of meiotic recombination, ultimately increasing genetic diversity within the population [2,33].

Indeed, microsatellite genotyping has revealed that the level of genetic variability is highly variable among distinct *P. vivax *populations worldwide. Using the same panel of 17 microsatellites, 100% of *P. vivax *infections in southern Vietnam were found to be polyclonal [33], compared to 11-70% polyclonality observed following analysis of isolates collected in the Peruvian Amazon [28] (Table 1). Similarly, when using a panel of nine microsatellites Imwong and colleagues reported low genetic diversity, high levels of inbreeding and linkage disequilibrium in Colombia, compared to high levels of genetic diversity in India, Thailand and Laos [27] (Table 1). These results emphasize that it cannot be assumed that global parasite populations are equivalent and as a result, may not be impacted by intervention measures to the same extent.

In order to enable accurate comparisons of genetic diversity of global *P. vivax *populations, a standardized approach to microsatellite genotyping is required, similar to that implemented for investigation of *P. falciparum *[67]. However, there remain a number of challenges and limitations to developing such an approach. Selection of both the appropriate number and length of microsatellites to be used for genotyping is crucial to obtain accurate results [29]. Increasing the number of markers investigated increases the likelihood of detecting multiple clone infections. Havryliuk and colleagues reported that a combination of nine markers was sufficient to identify 90% of multiple clone infections amongst samples collected in Acre, Brazil and that 11 markers enabled 100% of multiple clone infections to be identified [25]. In addition, it is known that repeat length can influence diversity associated with a microsatellite, with longer arrays of di-, tri- and tetra-nucleotide repeats more diverse than shorter sequences [2,53,68-71].

The diversity of a given microsatellite, and the number of microsatellites required to accurately genotype *P. vivax *strains will differ in different epidemiological settings and geographic regions [27,33,63]. Gunawardena and colleagues reported diversity of the MS16 microsatellite was far higher amongst Sri Lankan *P. vivax *strains compared to strains collected in Ethiopia, despite a higher rate of polyclonal infections detected amongst the Ethiopian samples tested [37] (Table 1). A similarly high level of MS16 diversity was observed by Koepfli and colleagues, reporting that more clones were detected using MS16, compared with the MSP1-f3 marker amongst strains from Papua New Guinea (PNG), due to a greater likelihood of clones sharing the same MSP1-f3 allele [72]. Furthermore, the same authors also demonstrated that in the context of a multiple clone infection, clones representing a minority of the population may be missed by PCR, and that detectability of specific *P. vivax *clones in a particular individual varied depending on the day that sample was collected [72]. Taken together, these results suggest that prior to development of a standardized strategy for *P. vivax *genotyping, the suitability of candidate markers must be widely assessed in distinct populations. In addition to identifying suitable markers, the criteria for assigning minor/multiple alleles must also be standardized to further limit discrepancies reported between studies.

## The importance of *Plasmodium vivax *population genetics studies

### Identifying routes of transmission and gene flow

Investigating the genetic diversity of *Plasmodium vivax *populations is essential from both a public health perspective, and

 to help achieve malaria control and elimination. Detection and analysis of individual clones within populations has not only provided a greater understanding of genetic diversity, but also of *P. vivax *prevalence and incidence [31,46,73,74]. Prevalence is one indicator of transmission and the main malariometric measurement in investigations including the global Malaria Atlas Project (MAP) [75]. For control and elimination strategies to succeed, the origin and movement of *P. vivax *populations must be identified, which can be achieved by genotyping isolates from several different regions. Using a panel of 12 microsatellite markers, Gunawardena and colleagues reported that isolates collected in Sri Lanka, Myanmar and Ethiopia all clustered according to their geographic origins, demonstrating that these microsatellites may be used to map the origin of *P. vivax *isolates, at least on a broad continental scale [37]. Genome-wide single nucleotide polymorphisms (SNPs) may provide a higher resolution for geographic positioning on a local scale, however such SNPs have not yet been identified for *P. vivax*.

The construction of population structure networks and maps demonstrating genetic relatedness of parasites in specific regions can enable not only the identification of parasite origins but also routes of population movement, and therefore the likelihood of successful elimination within a given region with respect to parasite diversity, population movement and the burden of imported cases [76]. The Pacific Malaria Initiative (PacMI) recently reported the results of an epidemiological survey performed in order to investigate the likelihood of success of potential malaria elimination campaigns in two isolated island provinces of Vanuatu and the Solomon Islands, Tafea and Temotu provinces, respectively [77]. The results of prior studies conducted in other provinces of both nations reported a predominance of *P. falciparum*, with high parasite prevalence (up to 35%) and transmission rates [77-80]. Although extensive genetic characterization of the parasite populations was not performed, the results of the recent PacMI survey revealed that compared to other provinces within each country, the malaria epidemiology in the isolated Tafea and Temotu provinces was hypoendemic, with low parasite prevalence and a predominance of *P. vivax *[77]. Coupled with restricted travel and screening of incoming passengers for infection with malaria parasites, both island provinces were flagged as candidates for future malaria elimination campaigns [77].

Porous land borders between malaria endemic countries, specifically throughout Southeast Asia and the Middle East, are potential barriers to the successful implementation of malaria elimination campaigns [77,81]. Migration of infected individuals resulting in importation of *Plasmodium *spp. and subsequent transmission by local vectors has contributed to the global spread of malaria [82]. Migration of infected individuals may seriously confound containment of *P. vivax*, as individuals can be unaware of their infection status due to long incubation periods. Hence, the potential for reintroduction of endemic malaria into non-endemic countries, is high. Khan and colleagues recently reported both detection of competent malaria vectors and an increase in malaria cases, mostly due to *P. vivax*, in Qatar, due to a massive influx of migrant workers from India and Pakistan [83]. Imported cases also have the potential to increase genetic diversity of existing *P. vivax *populations in malaria endemic countries [82]. Following reports of increasing *P. vivax *genetic diversity in Korea [84], Choi and colleagues used genotyping to successfully distinguish between autochthonous and imported *P. vivax *cases, identifying that the imported infections originated in neighbouring Asian countries. Imported infections also pose a risk in non-endemic regions of endemic countries. Many South American countries are geographically heterogeneous with regard to malaria transmission, and imported cases can result in serious illness and death amongst non-immune populations [85].

Genotyping imported infections is also essential to monitor the introduction and spread of drug resistance into sensitive populations. Population genetic surveys in Venezuela enabled identification of the introduction and spread of chloroquine resistant *P. falciparum*, resulting in changes to the national malaria treatment guidelines and rollout of more effective anti-malarials to combat the spread of drug resistance [82].

Combined, the results of these studies demonstrate the importance of population surveillance; to enable identification of routes of *P. vivax *introduction to, and transmission within, a given population. To achieve malaria elimination targets, the movement of *P. vivax *populations must be controlled to limit not only reintroduction of the disease into non-endemic countries, but also to restrict global genetic diversity and the spread of drug resistance.

## Impact assessment of intervention and vector control strategies

Investigation of genetic diversity within *P. vivax *populations is a useful gauge of both the likelihood of success and subsequently, the impact of intervention methods. Interventions such as anti-malarials and candidate vaccines would be anticipated to be more successful in a population with low genetic diversity and any reduction in genetic diversity following the introduction of intervention measures may be regarded as an indicator of success.

In the absence of a continuous *in vitro *culture system and defined markers to identify drug resistance, genotyping is currently used to monitor treatment efficacy, and the emergence of resistant *P. vivax *strains. An understanding of haplotype frequency within a given population is therefore essential [86]. With respect to *P. vivax *parasites detected following drug treatment, there are three possible sources: re-infection with a new clone, recrudescence of a drug resistant clone, or relapse as a result of reactivation of liver hypnozoites [87]. As biomarkers do not currently exist to determine whether recurrent *P. vivax *parasitaemia is due to reactivation of liver hypnozoites, genotyping is used to identify whether recurrent episodes of *P. vivax *infection are the result of re-infection with a new clone or recrudescence/relapse of an existing drug-resistant clone [87,88]. The basis for this approach is that genetic diversity is sufficient to be able to distinguish between strains using a panel of diverse markers [86]. Confounding the distinction between relapse and re-infection, Imwong and colleagues reported that contrary to the long-held belief that reactivated hypnozoites were genetically identical to the strain responsible for primary infection, reactivated hypnozoites might indeed be heterologous [87]. Hence, it may not always be possible to distinguish re-infection from relapse, especially in regions with reduced parasite diversity. To maximize the ability to distinguish between strains, a panel of diverse markers should be used [63,86,89]. The markers used must be sufficiently diverse and located in distinct genomic positions, however markers may be more or less suited for use dependent upon genetic diversity within a given population [27,63,86]. As a result, community-based investigations of parasite diversity and allele frequencies are vital to enable accurate analysis of anti-malarial interventions [27,86].

The impact of vector control strategies, such as insecticide spraying, is also measurable using population genetics methods. Jongwutiwes and colleagues reported differences in the diversity of *P. vivax *amongst populations in the north-west and south of Thailand [90]. Limited diversity, suggestive of a recent population bottleneck was observed in the south, where anti-malarial insecticide spraying had been implemented and was ongoing. In the north-west, a region bordering Myanmar, anti-malarial control measures have not been implemented to the same extent as in the south, and as a result, diversity of the *P. vivax *population investigated was high [90].

## Identification of immunogenic targets and potential vaccine candidates

Development of a vaccine targeting *P. vivax *lags far behind efforts to design a vaccine against *P. falciparum *[91]. This is an inevitable reflection of the reduced research focus on *P. vivax*. The main obstacle impeding *P. vivax *research is the lack of available parasite material, since *P. vivax *cannot be continuously cultured *in vitro *and infected individuals typically present with low parasitaemia [92-95]. As a result, the majority of clinical immunology studies rely on using recombinant *P. vivax *proteins, typically expressed from reference strains [95]. However, an understanding of population genetic structure can also give insight into the development of host immune responses. Population genetics studies can identify signatures of balancing selection within parasite surface antigen genes, enabling identification of domains targeted by strong host immune pressure and thus identification of potential vaccine candidates, as has been done for *P. falciparum *[23,96,97]. The utility of diversity data is enhanced when additional information is known, such as the allelic frequency within a given population [96]. For example, in a population with low microsatellite diversity, low diversity amongst genes encoding antigens would also be expected. As strain-specific immunity is thought to be a major reason for the failure of malaria vaccine trials to date [98], reduced diversity amongst antigen-encoding genes would encode less diverse surface antigens, increasing the breadth of vaccine efficacy, and the generation of effective immune responses [99,100].

There are three phases of the malaria parasite lifecycle that could be effectively targeted by host immune responses: inhibition of hepatocyte invasion at the pre-erythrocytic stage (e.g. vaccines targeting antigens such as CSP), inhibition of erythrocyte invasion during the asexual blood stage (merozoite surface protein 1, MSP1; apical membrane antigen 1, AMA1), and inhibition of parasite fertilization and development in the mosquito midgut (oocyst/ookinete 25 kD surface protein, Pvs25) [91,101]. The majority of the vaccine candidates currently under investigation for *P. vivax *are orthologues of *P. falciparum *vaccine candidate antigens [91,101]. However, due to biological differences, and differences in the extent and distribution of genetic diversity, it is not always possible to draw conclusions for *P. vivax *on the basis of what is known for *P. falciparum*. Few studies have been performed to investigate the diversity of vaccine candidate antigens in sympatric populations. This is despite the fact that many believe a globally effective malaria vaccine must contain not only multiple antigens, but also a combination of *P. falciparum *and *P. vivax *antigens due to sympatric circulation of both species in many endemic regions [101]. Indeed, differences may exist between regions of the same antigen under immune pressure in *P. falciparum *and *P. vivax *parasites, as has been reported for AMA1 [97,102-104]. Assessment of genetic diversity, and therefore suitability of candidate antigens is therefore essential to design an effective multi-species and/or a *P. vivax *vaccine.

## Conclusions

Partly as a consequence of the common misconception that *P. vivax *infection is benign, research funding and focus directed towards this neglected malaria parasite has been limited relative to *P. falciparum*. As a result, much remains unknown regarding *P. vivax *biology, epidemiology and pathogenesis. However, it is now understood that globally, *P. vivax *populations are highly genetically diverse, and that this diversity varies greatly according to geographic region. In order to achieve malaria control and elimination targets, population genetic surveys are vital to map the diversity and structure of local populations and to estimate the likelihood of success and measure the outcome of malaria intervention methods. To assist vaccine development, genetic structure and diversity of candidate antigens needs to be assessed in sympatric *P. vivax *and *P. falciparum *populations worldwide.

## Competing interests

The authors declare that they have no competing interests.

## Authors' contributions

AA drafted the paper. AEB and JCR provided input into scope and content and assisted in drafting the paper. All authors read and approved the final manuscript.

## References

[B1] Guerra CA Howes RE Patil AP Gething PW Van Boeckel TP Temperley WH Kabaria CW Tatem AJ Manh BH Elyazar IR Baird JK Snow RW Hay SI The international limits and population at risk of *Plasmodium vivax *transmission in 2009 PLoS Negl Trop Dis 2010 4 e774 10.1371/journal.pntd.0000774 20689816 PMC2914753

[B2] Ferreira MU Karunaweera ND da Silva-Nunes M da Silva NS Wirth DF Hartl DL Population structure and transmission dynamics of *Plasmodium vivax *in rural Amazonia J Infect Dis 2007 195 1218 1226 10.1086/512685 17357061

[B3] Kasehagen LJ Mueller I McNamara DT Bockarie MJ Kiniboro B Rare L Lorry K Kastens W Reeder JC Kazura JW Zimmerman PA Changing patterns of Plasmodium blood-stage infections in the Wosera region of Papua New Guinea monitored by light microscopy and high throughput PCR diagnosis Am J Trop Med Hyg 2006 75 588 596 17038678 PMC3728901

[B4] Jun G Yeom JS Hong JY Shin EH Chang KS Yu JR Oh S Chung H Park JW Resurgence of *Plasmodium vivax *malaria in the Republic of Korea during 2006-2007 Am J Trop Med Hyg 2009 81 605 610 10.4269/ajtmh.2009.09-0111 19815874

[B5] Carme B Ardillon V Girod R Grenier C Joubert M Djossou F Ravachol F Update on the epidemiology of malaria in French Guiana Med Trop (Mars) 2009 69 19 25 19499726

[B6] Mendis K Sina BJ Marchesini P Carter R The neglected burden of *Plasmodium vivax *malaria Am J Trop Med Hyg 2001 64 97 106 11425182 10.4269/ajtmh.2001.64.97

[B7] Price RN Tjitra E Guerra CA Yeung S White NJ Anstey NM Vivax malaria: neglected and not benign Am J Trop Med Hyg 2007 77 79 87 18165478 PMC2653940

[B8] Mueller I Galinski MR Baird JK Carlton JM Kochar DK Alonso PL del Portillo HA Key gaps in the knowledge of *Plasmodium vivax*, a neglected human malaria parasite Lancet Infect Dis 2009 9 555 566 10.1016/S1473-3099(09)70177-X 19695492

[B9] Genton B D'Acremont V Rare L Baea K Reeder JC Alpers MP Muller I *Plasmodium vivax *and mixed infections are associated with severe malaria in children: a prospective cohort study from Papua New Guinea PLoS Med 2008 5 e127 10.1371/journal.pmed.0050127 18563961 PMC2429951

[B10] Tjitra E Anstey NM Sugiarto P Warikar N Kenangalem E Karyana M Lampah DA Price RN Multidrug-resistant *Plasmodium vivax *associated with severe and fatal malaria: a prospective study in Papua, Indonesia PLoS Med 2008 5 e128 10.1371/journal.pmed.0050128 18563962 PMC2429950

[B11] Alexandre MA Ferreira CO Siqueira AM Magalhaes BL Mourao MP Lacerda MV Alecrim MG Severe *Plasmodium vivax *malaria, Brazilian Amazon Emerg Infect Dis 2010 16 1611 1614 20875292 10.3201/eid1610.100685 PMC3294402

[B12] Barcus MJ Basri H Picarima H Manyakori C Sekartuti Elyazar I Bangs MJ Maguire JD Baird JK Demographic risk factors for severe and fatal vivax and falciparum malaria among hospital admissions in northeastern Indonesian Papua Am J Trop Med Hyg 2007 77 984 991 17984364

[B13] Fernandez-Becerra C Pinazo MJ Gonzalez A Alonso PL del Portillo HA Gascon J Increased expression levels of the pvcrt-o and pvmdr1 genes in a patient with severe *Plasmodium vivax *malaria Malar J 2009 8 55 10.1186/1475-2875-8-55 19341456 PMC2682795

[B14] Kochar DK Das A Kochar SK Saxena V Sirohi P Garg S Kochar A Khatri MP Gupta V Severe *Plasmodium vivax *malaria: a report on serial cases from Bikaner in northwestern India Am J Trop Med Hyg 2009 80 194 198 19190212

[B15] Feachem RGA, Phillips AA, Targett GA Shrinking the Malaria Map: A Prospectus on Malaria Elimination 2009 First San Francisco: The Global Health Group, Global Health Sciences, University of California, San Francisco

[B16] The maIERA Consultative Group on Drugs A research agenda for malaria eradication: drugs PLoS Med 2011 8 e1000402 21311580 10.1371/journal.pmed.1000402 PMC3026688

[B17] Baird JK Hoffman SL Primaquine therapy for malaria Clin Infect Dis 2004 39 1336 1345 10.1086/424663 15494911

[B18] Joshi H Prajapati SK Verma A Kang'a S Carlton JM *Plasmodium vivax *in India Trends Parasitol 2008 24 228 235 10.1016/j.pt.2008.01.007 18403267

[B19] Schultz L Wapling J Mueller I Ntsuke PO Senn N Nale J Kiniboro B Buckee CO Tavul L Siba PM Reeder JC Barry AE Multilocus haplotypes reveal variable levels of diversity and population structure of *Plasmodium falciparum *in Papua New Guinea, a region of intense perennial transmission Malar J 2010 9 336 10.1186/1475-2875-9-336 21092231 PMC3002378

[B20] Joy DA Gonzalez-Ceron L Carlton JM Gueye A Fay M McCutchan TF Su XZ Local adaptation and vector-mediated population structure in *Plasmodium vivax *malaria Mol Biol Evol 2008 25 1245 1252 10.1093/molbev/msn073 18385220 PMC2386084

[B21] Reid H Vallely A Taleo G Tatem AJ Kelly G Riley I Harris I Henri I Iamaher S Clements AC Baseline spatial distribution of malaria prior to an elimination programme in Vanuatu Malar J 2010 9 150 10.1186/1475-2875-9-150 20525209 PMC2893196

[B22] Grynberg P Fontes CJ Hughes AL Braga EM Polymorphism at the apical membrane antigen 1 locus reflects the world population history of *Plasmodium vivax* BMC Evol Biol 2008 8 123 10.1186/1471-2148-8-123 18445274 PMC2394524

[B23] Cui L Escalante AA Imwong M Snounou G The genetic diversity of *Plasmodium vivax *populations Trends Parasitol 2003 19 220 226 10.1016/S1471-4922(03)00085-0 12763428

[B24] Baum J Billker O Bousema T Dinglasan R McGovern V Mota MM Mueller I Sinden R A research agenda for malaria eradication: basic science and enabling technologies PLoS Med 2011 8 e1000399 21311584 10.1371/journal.pmed.1000399 PMC3026698

[B25] Havryliuk T Ferreira MU A closer look at multiple-clone *Plasmodium vivax *infections: detection methods, prevalence and consequences Mem Inst Oswaldo Cruz 2009 104 67 73 19274379 10.1590/s0074-02762009000100011

[B26] Karunaweera ND Ferreira MU Munasinghe A Barnwell JW Collins WE King CL Kawamoto F Hartl DL Wirth DF Extensive microsatellite diversity in the human malaria parasite *Plasmodium vivax* Gene 2008 410 105 112 10.1016/j.gene.2007.11.022 18226474

[B27] Imwong M Nair S Pukrittayakamee S Sudimack D Williams JT Mayxay M Newton PN Kim JR Nandy A Osorio L Carlton JM White NJ Day NP Anderson TJ Contrasting genetic structure in *Plasmodium vivax *populations from Asia and South America Int J Parasitol 2007 37 1013 1022 10.1016/j.ijpara.2007.02.010 17442318

[B28] Van den Eede P Van der Auwera G Delgado C Huyse T Soto-Calle VE Gamboa D Grande T Rodriguez H Llanos A Anne J Erhart A D'Alessandro U Multilocus genotyping reveals high heterogeneity and strong local population structure of the *Plasmodium vivax *population in the Peruvian Amazon Malar J 2010 9 151 10.1186/1475-2875-9-151 20525233 PMC2898784

[B29] Brito CF Ferreira MU Molecular markers and genetic diversity of *Plasmodium vivax* Mem Inst Oswaldo Cruz 2011 106 (Suppl 1):12 26 10.1590/s0074-02762011000900003 21881753

[B30] Imwong M Pukrittayakamee S Gruner AC Renia L Letourneur F Looareesuwan S White NJ Snounou G Practical PCR genotyping protocols for *Plasmodium vivax *using Pvcs and Pvmsp1 Malar J 2005 4 20 10.1186/1475-2875-4-20 15854233 PMC1131918

[B31] Cui L Mascorro CN Fan Q Rzomp KA Khuntirat B Zhou G Chen H Yan G Sattabongkot J Genetic diversity and multiple infections of *Plasmodium vivax *malaria in Western Thailand Am J Trop Med Hyg 2003 68 613 619 12812356 10.4269/ajtmh.2003.68.613

[B32] Rungsihirunrat K Chaijaroenkul W Siripoon N Seugorn A Na-Bangchang K Genotyping of polymorphic marker (MSP3alpha and MSP3beta) genes of *Plasmodium vivax *field isolates from malaria endemic of Thailand Trop Med Int Health 2011 16 794 801 10.1111/j.1365-3156.2011.02771.x 21447062

[B33] Van den Eede P Erhart A Van der Auwera G Van Overmeir C Thang ND Hung le X Anne J D'Alessandro U High complexity of *Plasmodium vivax *infections in symptomatic patients from a rural community in central Vietnam detected by microsatellite genotyping Am J Trop Med Hyg 2010 82 223 227 10.4269/ajtmh.2010.09-0458 20133996 PMC2813161

[B34] Prajapati SK Verma A Adak T Yadav RS Kumar A Eapen A Das MK Singh N Sharma SK Rizvi MA Dash AP Joshi H Allelic dimorphism of *Plasmodium vivax *gam-1 in the Indian subcontinent Malar J 2006 5 90 10.1186/1475-2875-5-90 17062127 PMC1630701

[B35] Kim JR Imwong M Nandy A Chotivanich K Nontprasert A Tonomsing N Maji A Addy M Day NP White NJ Pukrittayakamee S Genetic diversity of *Plasmodium vivax *in Kolkata, India Malar J 2006 5 71 10.1186/1475-2875-5-71 16907979 PMC1560144

[B36] Wickramarachchi T Premaratne PH Dias S Handunnetti SM Udagama-Randeniya PV Genetic complexity of *Plasmodium vivax *infections in Sri Lanka, as reflected at the merozoite-surface-protein-3alpha locus Ann Trop Med Parasitol 2010 104 95 108 10.1179/136485910X12607012374190 20406577

[B37] Gunawardena S Karunaweera ND Ferreira MU Phone-Kyaw M Pollack RJ Alifrangis M Rajakaruna RS Konradsen F Amerasinghe PH Schousboe ML Galappaththy GN Abeyasinghe RR Hartl DL Wirth DF Geographic structure of *Plasmodium vivax*: microsatellite analysis of parasite populations from Sri Lanka, Myanmar, and Ethiopia Am J Trop Med Hyg 2010 82 235 242 10.4269/ajtmh.2010.09-0588 20133999 PMC2813164

[B38] Khatoon L Baliraine FN Bonizzoni M Malik SA Yan G Genetic structure of *Plasmodium vivax *and *Plasmodium falciparum *in the Bannu district of Pakistan Malar J 2010 9 112 10.1186/1475-2875-9-112 20416089 PMC2873525

[B39] Zakeri S Raeisi A Afsharpad M Kakar Q Ghasemi F Atta H Zamani G Memon MS Salehi M Djadid ND Molecular characterization of *Plasmodium vivax *clinical isolates in Pakistan and Iran using pvmsp-1, pvmsp-3alpha and pvcsp genes as molecular markers Parasitol Int 2010 59 15 21 10.1016/j.parint.2009.06.006 19545647

[B40] Yang Z Miao J Huang Y Li X Putaporntip C Jongwutiwes S Gao Q Udomsangpetch R Sattabongkot J Cui L Genetic structures of geographically distinct *Plasmodium vivax *populations assessed by PCR/RFLP analysis of the merozoite surface protein 3beta gene Acta Trop 2006 100 205 212 10.1016/j.actatropica.2006.10.011 17129568 PMC1839953

[B41] Zhong D Bonizzoni M Zhou G Wang G Chen B Vardo-Zalik A Cui L Yan G Zheng B Genetic diversity of *Plasmodium vivax *malaria in China and Myanmar Infect Genet Evol 2011 11 1419 1425 10.1016/j.meegid.2011.05.009 21624503 PMC3143071

[B42] Kim TS Kim HH Lee SS Na BK Lin K Cho SH Kang YJ Kim DK Sohn Y Kim H Lee HW Prevalence of *Plasmodium vivax *VK210 and VK247 subtype in Myanmar Malar J 2010 9 195 10.1186/1475-2875-9-195 20615261 PMC2914060

[B43] Moon SU Lee HW Kim JY Na BK Cho SH Lin K Sohn WM Kim TS High frequency of genetic diversity of *Plasmodium vivax *field isolates in Myanmar Acta Trop 2009 109 30 36 10.1016/j.actatropica.2008.09.006 18851938

[B44] Chen N Auliff A Rieckmann K Gatton M Cheng Q Relapses of *Plasmodium vivax *infection result from clonal hypnozoites activated at predetermined intervals J Infect Dis 2007 195 934 941 10.1086/512242 17330782

[B45] Zakeri S Dinparast Djadid N Zeinali S Sequence heterogeneity of the merozoite surface protein-1 gene (MSP-1) of *Plasmodium vivax *wild isolates in southeastern Iran Acta Trop 2003 88 91 97 10.1016/S0001-706X(03)00192-X 12943983

[B46] Zakeri S Abouie Mehrizi A Djadid ND Snounou G Circumsporozoite protein gene diversity among temperate and tropical *Plasmodium vivax *isolates from Iran Trop Med Int Health 2006 11 729 737 10.1111/j.1365-3156.2006.01613.x 16640626

[B47] Zakeri S Barjesteh H Djadid ND Merozoite surface protein-3alpha is a reliable marker for population genetic analysis of *Plasmodium vivax* Malar J 2006 5 53 10.1186/1475-2875-5-53 16817951 PMC1579225

[B48] Severini C Menegon M Di Luca M Abdullaev I Majori G Razakov SA Gradoni L Risk of *Plasmodium vivax *malaria reintroduction in Uzbekistan: genetic characterization of parasites and status of potential malaria vectors in the Surkhandarya region Trans R Soc Trop Med Hyg 2004 98 585 592 10.1016/j.trstmh.2004.01.003 15289095

[B49] Zakeri S Safi N Afsharpad M Butt W Ghasemi F Mehrizi AA Atta H Zamani G Djadid ND Genetic structure of *Plasmodium vivax *isolates from two malaria endemic areas in Afghanistan Acta Trop 2010 113 12 19 10.1016/j.actatropica.2009.08.025 19716798

[B50] Zeyrek FY Tachibana S Yuksel F Doni N Palacpac N Arisue N Horii T Coban C Tanabe K Limited polymorphism of the *Plasmodium vivax *merozoite surface protein 1 gene in isolates from Turkey Am J Trop Med Hyg 2010 83 1230 1237 10.4269/ajtmh.2010.10-0353 21118926 PMC2990036

[B51] Sutton PL Neyra V Hernandez JN Branch OH *Plasmodium falciparum *and *Plasmodium vivax *infections in the Peruvian Amazon: propagation of complex, multiple allele-type infections without super-infection Am J Trop Med Hyg 2009 81 950 960 10.4269/ajtmh.2009.09-0132 19996422 PMC3773727

[B52] Rezende AM Tarazona-Santos E Couto AD Fontes CJ De Souza JM Carvalho LH Brito CF Analysis of genetic variability of *Plasmodium vivax *isolates from different Brazilian Amazon areas using tandem repeats Am J Trop Med Hyg 2009 80 729 733 19407114

[B53] Rezende AM Tarazona-Santos E Fontes CJ Souza JM Couto AD Carvalho LH Brito CF Microsatellite loci: determining the genetic variability of *Plasmodium vivax* Trop Med Int Health 2010 15 718 726 10.1111/j.1365-3156.2010.02535.x 20406424

[B54] Storti-Melo LM de Souza-Neiras WC Cassiano GC Joazeiro AC Fontes CJ Bonini-Domingos CR Couto AA Povoa MM de Mattos LC Cavasini CE Rossit AR Machado RL *Plasmodium vivax *circumsporozoite variants and Duffy blood group genotypes in the Brazilian Amazon region Trans R Soc Trop Med Hyg 2009 103 672 678 10.1016/j.trstmh.2008.07.018 18804827

[B55] Orjuela-Sanchez P da Silva NS da Silva-Nunes M Ferreira MU Recurrent parasitemias and population dynamics of *Plasmodium vivax *polymorphisms in rural Amazonia Am J Trop Med Hyg 2009 81 961 968 10.4269/ajtmh.2009.09-0337 19996423

[B56] Veron V Legrand E Yrinesi J Volney B Simon S Carme B Genetic diversity of msp3alpha and msp1_b5 markers of *Plasmodium vivax *in French Guiana Malar J 2009 8 40 10.1186/1475-2875-8-40 19284592 PMC2660359

[B57] Bonilla JA Validum L Cummings R Palmer CJ Genetic diversity of *Plasmodium vivax *Pvcsp and Pvmsp1 in Guyana, South America Am J Trop Med Hyg 2006 75 830 835 17123973

[B58] Cristiano FA Perez MA Nicholls RS Guerra AP Polymorphism in the *Plasmodium vivax *msp 3: gene in field samples from Tierralta, Colombia Mem Inst Oswaldo Cruz 2008 103 493 496 10.1590/S0074-02762008000500015 18797765

[B59] Ord R Polley S Tami A Sutherland CJ High sequence diversity and evidence of balancing selection in the Pvmsp3alpha gene of *Plasmodium vivax *in the Venezuelan Amazon Mol Biochem Parasitol 2005 144 86 93 10.1016/j.molbiopara.2005.08.005 16159677

[B60] Leclerc MC Gauthier C Villegas L Urdaneta L Genetic diversity of merozoite surface protein-1 gene of *Plasmodium vivax *isolates in mining villages of Venezuela (Bolivar State) Acta Trop 2005 95 26 32 10.1016/j.actatropica.2005.03.007 15862584

[B61] Henry-Halldin CN Sepe D Susapu M McNamara DT Bockarie M King CL Zimmerman PA High-throughput molecular diagnosis of circumsporozoite variants VK210 and VK247 detects complex *Plasmodium vivax *infections in malaria endemic populations in Papua New Guinea Infect Genet Evol 2011 11 391 398 10.1016/j.meegid.2010.11.010 21147267 PMC3728899

[B62] Mueller I Kaiok J Reeder JC Cortes A The population structure of *Plasmodium falciparum *and *Plasmodium vivax *during an epidemic of malaria in the Eastern Highlands of Papua New Guinea Am J Trop Med Hyg 2002 67 459 464 12479544 10.4269/ajtmh.2002.67.459

[B63] Koepfli C Mueller I Marfurt J Goroti M Sie A Oa O Genton B Beck HP Felger I Evaluation of *Plasmodium vivax *genotyping markers for molecular monitoring in clinical trials J Infect Dis 2009 199 1074 1080 10.1086/597303 19275476

[B64] Gomez JC McNamara DT Bockarie MJ Baird JK Carlton JM Zimmerman PA Identification of a polymorphic *Plasmodium vivax *microsatellite marker Am J Trop Med Hyg 2003 69 377 379 14640496 PMC3728893

[B65] Anderson TJ Haubold B Williams JT Estrada-Franco JG Richardson L Mollinedo R Bockarie M Mokili J Mharakurwa S French N Whitworth J Velez ID Brockman AH Nosten F Ferreira MU Day KP Microsatellite markers reveal a spectrum of population structures in the malaria parasite *Plasmodium falciparum* Mol Biol Evol 2000 17 1467 1482 11018154 10.1093/oxfordjournals.molbev.a026247

[B66] Mu J Awadalla P Duan J McGee KM Joy DA McVean GA Su XZ Recombination hotspots and population structure in *Plasmodium falciparum* PLoS Biol 2005 3 e335 10.1371/journal.pbio.0030335 16144426 PMC1201364

[B67] Anderson TJ Su XZ Bockarie M Lagog M Day KP Twelve microsatellite markers for characterization of *Plasmodium falciparum *from finger-prick blood samples Parasitology 1999 119 Pt 2 113 125 10466118 10.1017/s0031182099004552

[B68] Imwong M Sudimack D Pukrittayakamee S Osorio L Carlton JM Day NP White NJ Anderson TJ Microsatellite variation, repeat array length, and population history of *Plasmodium vivax* Mol Biol Evol 2006 23 1016 1018 10.1093/molbev/msj116 16507919

[B69] Russell B Suwanarusk R Lek-Uthai U *Plasmodium vivax *genetic diversity: microsatellite length matters Trends Parasitol 2006 22 399 401 10.1016/j.pt.2006.06.013 16837246

[B70] Leclerc MC Durand P Gauthier C Patot S Billotte N Menegon M Severini C Ayala FJ Renaud F Meager genetic variability of the human malaria agent *Plasmodium vivax* Proc Natl Acad Sci USA 2004 101 14455 14460 10.1073/pnas.0405186101 15328406 PMC521958

[B71] de Souza-Neiras WC de Melo LM Machado RL The genetic diversity of *Plasmodium vivax*--a review Mem Inst Oswaldo Cruz 2007 102 245 254 17568928 10.1590/s0074-02762007000300002

[B72] Koepfli C Schoepflin S Bretscher M Lin E Kiniboro B Zimmerman PA Siba P Smith TA Mueller I Felger I How much remains undetected? Probability of molecular detection of human plasmodia in the field PLoS One 2011 6 e19010 10.1371/journal.pone.0019010 21552561 PMC3084249

[B73] Bruce MC Galinski MR Barnwell JW Donnelly CA Walmsley M Alpers MP Walliker D Day KP Genetic diversity and dynamics of *plasmodium falciparum *and *P. vivax *populations in multiply infected children with asymptomatic malaria infections in Papua New Guinea Parasitology 2000 121 Pt 3 257 272 11085246 10.1017/s0031182099006356

[B74] Daubersies P Sallenave-Sales S Magne S Trape JF Contamin H Fandeur T Rogier C Mercereau-Puijalon O Druilhe P Rapid turnover of *Plasmodium falciparum *populations in asymptomatic individuals living in a high transmission area Am J Trop Med Hyg 1996 54 18 26 8651363 10.4269/ajtmh.1996.54.18

[B75] Guerra CA Hay SI Lucioparedes LS Gikandi PW Tatem AJ Noor AM Snow RW Assembling a global database of malaria parasite prevalence for the Malaria Atlas Project Malar J 2007 6 17 10.1186/1475-2875-6-17 17306022 PMC1805762

[B76] Moonen B Cohen JM Snow RW Slutsker L Drakeley C Smith DL Abeyasinghe RR Rodriguez MH Maharaj R Tanner M Targett G Operational strategies to achieve and maintain malaria elimination Lancet 2010 376 1592 1603 10.1016/S0140-6736(10)61269-X 21035841 PMC3037542

[B77] The Pacific Malaria Initiative Survey Group (PMISG) on behalf of the Ministries of Health of Vanuatu and Solomon Islands Malaria on isolated Melanesian islands prior to the initiation of malaria elimination activities Malar J 2010 9 218 20659316 10.1186/1475-2875-9-218 PMC2921077

[B78] Maguire JD Bangs MJ Brennan L Rieckmann K Taleo G Cross-sectional characterization of malaria in Sanma and Shefa Provinces, Republic of Vanuatu: malaria control implications P N G Med J 2006 49 22 31 18396609

[B79] Maitland K Williams TN Peto TE Day KP Clegg JB Weatherall DJ Bowden DK Absence of malaria-specific mortality in children in an area of hyperendemic malaria Trans R Soc Trop Med Hyg 1997 91 562 566 10.1016/S0035-9203(97)90026-2 9463668

[B80] Hii JL Kanai L Foligela A Kan SK Burkot TR Wirtz RA Impact of permethrin-impregnated mosquito nets compared with DDT house-spraying against malaria transmission by Anopheles farauti and An.punctulatus in the Solomon Islands Med Vet Entomol 1993 7 333 338 10.1111/j.1365-2915.1993.tb00701.x 8268487

[B81] Feachem R Sabot O A new global malaria eradication strategy Lancet 2008 371 1633 1635 10.1016/S0140-6736(08)60424-9 18374409

[B82] Rodriguez-Morales AJ Delgado L Martinez N Franco-Paredes C Impact of imported malaria on the burden of disease in northeastern Venezuela J Travel Med 2006 13 15 20 10.1111/j.1708-8305.2006.00006.x 16412105

[B83] Khan FY Lutof AK Yassin MA Khattab MA Saleh M Rezeq HY Almaslamani M Imported malaria in Qatar: a 1 year hospital-based study in 2005 Travel Med Infect Dis 2009 7 111 117 10.1016/j.tmaid.2009.01.003 19237144

[B84] Choi KM Choi YK Kang YA Seo SY Lee HW Cho SH Lee WJ Rhie HG Lee HS Kim JY Study of the genetic discrimination between imported and autochthonous cases of malaria in South Korea J Travel Med 2011 18 63 66 10.1111/j.1708-8305.2010.00473.x 21199147

[B85] Rodriguez-Morales AJ Ferrer MV Barrera MA Pacheco M Daza V Franco-Paredes C Imported cases of malaria admitted to two hospitals of Margarita Island, Venezuela, 1998-2005 Travel Med Infect Dis 2009 7 44 48 10.1016/j.tmaid.2008.09.006 19174301

[B86] Gatton ML Cheng Q Can estimates of antimalarial efficacy from field studies be improved? Trends Parasitol 2008 24 68 73 10.1016/j.pt.2007.11.003 18182325 PMC2646162

[B87] Imwong M Snounou G Pukrittayakamee S Tanomsing N Kim JR Nandy A Guthmann JP Nosten F Carlton J Looareesuwan S Nair S Sudimack D Day NP Anderson TJ White NJ Relapses of *Plasmodium vivax *infection usually result from activation of heterologous hypnozoites J Infect Dis 2007 195 927 933 10.1086/512241 17330781

[B88] Teka H Petros B Yamuah L Tesfaye G Elhassan I Muchohi S Kokwaro G Aseffa A Engers H Chloroquine-resistant *Plasmodium vivax *malaria in Debre Zeit, Ethiopia Malar J 2008 7 220 10.1186/1475-2875-7-220 18959774 PMC2584068

[B89] Havryliuk T Orjuela-Sanchez P Ferreira MU *Plasmodium vivax*: microsatellite analysis of multiple-clone infections Exp Parasitol 2008 120 330 336 10.1016/j.exppara.2008.08.012 18801362

[B90] Jongwutiwes S Putaporntip C Hughes AL Bottleneck effects on vaccine-candidate antigen diversity of malaria parasites in Thailand Vaccine 2010 28 3112 3117 10.1016/j.vaccine.2010.02.062 20199765 PMC2847032

[B91] Galinski MR Barnwell JW Plasmodium vivax: who cares? Malar J 2008 7 Suppl 1 S9 10.1186/1475-2875-7-S1-S9 19091043 PMC2604873

[B92] Golenda CF Li J Rosenberg R Continuous in vitro propagation of the malaria parasite *Plasmodium vivax* Proc Natl Acad Sci USA 1997 94 6786 6791 10.1073/pnas.94.13.6786 9192643 PMC21236

[B93] Mons B Collins WE Skinner JC van der Star W Croon JJ van der Kaay HJ *Plasmodium vivax*: in vitro growth and reinvasion in red blood cells of Aotus nancymai Exp Parasitol 1988 66 183 188 10.1016/0014-4894(88)90089-6 3294025

[B94] Chotivanich K Silamut K Udomsangpetch R Stepniewska KA Pukrittayakamee S Looareesuwan S White NJ Ex-vivo short-term culture and developmental assessment of *Plasmodium vivax* Trans R Soc Trop Med Hyg 2001 95 677 680 10.1016/S0035-9203(01)90113-0 11816444

[B95] Gentil F Bargieri DY Leite JA Francoso KS Patricio MB Espindola NM Vaz AJ Palatnik-de-Sousa CB Rodrigues MM Costa FT Soares IS A recombinant vaccine based on domain II of *Plasmodium vivax *Apical Membrane Antigen 1 induces high antibody titres in mice Vaccine 2010 28 6183 6190 10.1016/j.vaccine.2010.07.017 20654667

[B96] Weedall GD Conway DJ Detecting signatures of balancing selection to identify targets of anti-parasite immunity Trends Parasitol 2010 26 363 369 10.1016/j.pt.2010.04.002 20466591

[B97] Gunasekera AM Wickramarachchi T Neafsey DE Ganguli I Perera L Premaratne PH Hartl D Handunnetti SM Udagama-Randeniya PV Wirth DF Genetic diversity and selection at the *Plasmodium vivax *apical membrane antigen-1 (PvAMA-1) locus in a Sri Lankan population Mol Biol Evol 2007 24 939 947 10.1093/molbev/msm013 17244598

[B98] Richards JS Beeson JG The future for blood-stage vaccines against malaria Immunol Cell Biol 2009 87 377 390 10.1038/icb.2009.27 19417768

[B99] Dias S Longacre S Escalante AA Udagama-Randeniya PV Genetic diversity and recombination at the C-terminal fragment of the merozoite surface protein-1 of *Plasmodium vivax *(PvMSP-1) in Sri Lanka Infect Genet Evol 2011 11 145 156 10.1016/j.meegid.2010.09.007 20933611

[B100] Ferreira MU da Silva Nunes M Wunderlich G Antigenic diversity and immune evasion by malaria parasites Clin Diagn Lab Immunol 2004 11 987 995 15539495 10.1128/CDLI.11.6.987-995.2004 PMC524792

[B101] Arevalo-Herrera M Chitnis C Herrera S Current status of *Plasmodium vivax *vaccine Hum Vaccin 2010 6 124 132 10.4161/hv.6.1.9931 20009526

[B102] Remarque EJ Faber BW Kocken CH Thomas AW Apical membrane antigen 1: a malaria vaccine candidate in review Trends Parasitol 2008 24 74 84 10.1016/j.pt.2007.12.002 18226584

[B103] Ord RL Tami A Sutherland CJ ama1 genes of sympatric *Plasmodium vivax *and *P*. *falciparum *from Venezuela differ significantly in genetic diversity and recombination frequency PLoS One 2008 3 e3366 10.1371/journal.pone.0003366 18846221 PMC2559863

[B104] Polley SD Chokejindachai W Conway DJ Allele frequency-based analyses robustly map sequence sites under balancing selection in a malaria vaccine candidate antigen Genetics 2003 165 555 561 14573469 10.1093/genetics/165.2.555 PMC1462796

